# Nurses’ experiences and preferences around shift patterns: A scoping review

**DOI:** 10.1371/journal.pone.0256300

**Published:** 2021-08-16

**Authors:** Ourega-Zoé Ejebu, Chiara Dall’Ora, Peter Griffiths

**Affiliations:** 1 School of Health Sciences, University of Southampton, Southampton, United Kingdom; 2 National Institute for Health Research Applied Research Collaboration (Wessex), Southampton, United Kingdom; Universiteit van Amsterdam, NETHERLANDS

## Abstract

**Objective:**

To explore the evidence on nurses’ experiences and preferences around shift patterns in the international literature.

**Data sources:**

Electronic databases (CINHAL, MEDLINE and Scopus) were searched to identify primary studies up to April 2021.

**Methods:**

Papers reporting qualitative or quantitative studies exploring the subjective experience and/or preferences of nurses around shift patterns were considered, with no restrictions on methods, date or setting. Key study features were extracted including setting, design and results. Findings were organised thematically by key features of shift work.

**Results:**

30 relevant papers were published between 1993 and 2021. They contained mostly qualitative studies where nurses reflected on their experience and preferences around shift patterns. The studies reported on three major aspects of shift work: shift work *per se* (i.e. the mere fact of working shift), shift length, and time of shift. Across all three aspects of shift work, nurses strive to deliver high quality of care despite facing intense working conditions, experiencing physical and mental fatigue or exhaustion. Preference for or adaptation to a specific shift pattern is facilitated when nurses are consulted before its implementation or have a certain autonomy to self-roster. Days off work tend to mitigate the adverse effects of working (short, long, early or night) shifts. How shift work and patterns impact on experiences and preferences seems to also vary according to nurses’ personal characteristics and circumstances (e.g. age, caring responsibilities, years of experience).

**Conclusions:**

Shift patterns are often organised in ways that are detrimental to nurses’ health and wellbeing, their job performance, and the patient care they provide. Further research should explore the extent to which nurses’ preferences are considered when choosing or being imposed shift work patterns. Research should also strive to better describe and address the constraints nurses face when it comes to choice around shift patterns.

## Introduction

Shift work is an established feature of working life for many hospital nurses, who work to provide 24-hour healthcare. Several directives and regulations influence how shift work is organised, including the European Working Time Directive of 2003 [1] and the US Fair Labor Standards Act of 1938 [2]. Such directives limits the maximum number of weekly hours or regulate the frequency of work breaks. Notwithstanding such regulations, shift work can be organised in a variety of ways, in terms of shift length, overtime, weekly hours, rotating and/or permanent schedules. How shift patterns are organised play a key role in factors influencing nurses’ wellbeing and performance, as well as patient outcomes and health systems’ productivity [3].

For instance, shift work may require nurses to work overnight causing adverse health effects, such as increased sleepiness at the end of the shift [4] or disturbed sleep [5]. Shift work schedules can also have unintended consequences depending on whether they are rotating or permanent. Working permanent night shifts is associated with higher long-term sickness absence rates in comparison to day-shifts only [6]. In the same vein, working as part of a rotating schedule is associated with increased levels of acute fatigue [7], errors [8] and higher risks of alcohol consumption [9]. These factors can in turn jeopardise the quality of care.

While a three-shift pattern with two 8-hour day shifts and a night shift remains common, long shifts of 12 hours or more as part of a two-shift system have become standard in many countries including Ireland, Poland, the USA and increasingly in the UK [10, 11]. Despite a number of claims that a two-shift system is more efficient, there is no evidence of productivity gain when working long shifts [12] and job dissatisfaction is higher among nurses working long shifts [11, 13, 14]. Working long shifts is also associated with nurses reporting reduced educational opportunities, fewer opportunities to discuss patient care [15], increased delayed or missed care [16] and higher (pneumonia) mortality rates [17] in comparison to shorter shifts. Nurses working long shifts are also more likely to experience burnout and report intention to leave in comparison to their counterparts [13, 14].

Despite such adverse outcomes, some literature suggest certain nurses prefer working long shifts, as evidenced by their higher job and schedule satisfaction, as well as their lower emotional exhaustion level [18]. Preference for long shifts is also attributed to improved work-life balance [19], higher number of days off and opportunities for greater continuity of care [20]. However, the mechanisms explaining such preferences, how nurses experience these shift patterns, and how these shift patterns interact with other aspects of their life remains unclear.

The evidence on nurses’ subjective experience and preferences around shift patterns has not been summarised, as quantitative studies reporting associations dominate the field. In these studies, adverse experiences are indirectly inferred from (for example) reported associations between shift patterns and burnout. The purpose of such quantitative studies is generally not to capture nurses’ perspectives. Yet, insights from nurses’ perspective are key to better understand mechanisms of preference and choice around shift patterns. Studies where nurses’ perspectives are directly obtained (rather than inferred by the researcher) could shed further light on the contradictions arising from the quantitative body of evidence. Nursing staff form the largest group in the health workforce, and comprehending their experience and preferences around shift patterns is key to effectively improve nursing working conditions, enhance nurses’ job satisfaction and increase quality of care. This review focusses only on nurses because the experience of shift work is specific to the occupation and the context specific [21]. Therefore, the aim of this review is to examine and summarise the extent, range and nature of research activity on nurses’ subjective experience and preferences around shift patterns.

## Methods

Because of our broad research question, we conducted a scoping review [22, 23], aiming to summarise existing evidence and highlight gaps.

### Search and inclusion/exclusion strategy

We searched CINAHL, Medline and Scopus with terms pertaining to nurses’ experience and preferences around shift patterns. Searches were undertaken up to April 2021. Table 1 provides a detailed list of the key terms that we used for the search. We limited our search to studies with an English language abstract. There was no restriction on the publication date to ensure the review of research was comprehensive.

**Table 1:** Search strategy for scoping review on nurses’ experience and preferences around shift pattern.

**CINHAL (EBSCO) (N = 1,225)**
S1 shift work
S2 work schedule
S3 shift pattern
S4 shift length
S5 S1 OR S2 OR S3 OR S4
S6 “nurse”
S7 health professionals
S8 S6 OR S7
S9 S5 AND S8
S10 “impact”
S11 “effect”
S12 “affect”
S13 “perception”
S14 “experience”
S15 “reaction”
S16 “prefer*”
S17 S10 OR S11 OR S12 OR S13 OR S14 OR S15 OR S16
S18 S9 AND S17 Limiters: English
S19 S9 AND S16 Limiters: English—Research Article
**Medline (Ovid) (N = 1,054)**
1. (shift adj4 work*).mp. [mp = title, abstract, original title, name of substance word, subject heading word, floating sub-heading word, keyword heading word, organism supplementary concept word, protocol supplementary concept word, rare disease supplementary concept word, unique identifier, synonyms]
2. work* schedule.mp.
3. shift pattern*.mp.
4. shift length.mp.
5. "Personnel Staffing and Scheduling"/
6. (shift or schedule).mp. [mp = title, abstract, original title, name of substance word, subject heading word, floating sub-heading word, keyword heading word, organism supplementary concept word, protocol supplementary concept word, rare disease supplementary concept word, unique identifier, synonyms]
7. 5 and 6
8. 1 or 2 or 3 or 4 or 7
9. nurse*.mp.
10. health professional.mp.
11. 9 or 10
12. impact.mp.
13. effect.mp.
14. affect.mp. or Affect/
15. perception.mp. or Perception/
16. experience.mp.
17. reaction*.mp.
18. preference.mp.
19. 12 or 13 or 14 or 15 or 16 or 17 or 18
20. 8 and 11 and 19
21. 20 and “Journal Article” [Publication Type]
**Scopus (Elsevier) (N = 1,127)**
((TITLE-ABS-KEY (“shift work”)) OR (TITLE-ABS-KEY (“work schedule”)) OR (TITLE-ABS-KEY (“shift pattern”)) OR (TITLE-ABS-KEY (“shift length”))) AND ((TITLE-ABS-KEY (“nurse”)) OR (TITLE-ABS-KEY (“health professional”))) AND ((TITLE-ABS-KEY (“impact”)) OR (TITLE-ABS-KEY (“effect”)) OR (TITLE-ABS-KEY (“affect”)) OR (TITLE-ABS-KEY (“perception”)) OR (TITLE-ABS-KEY (“experience”)) OR (TITLE-ABS-KEY (“reaction”)) OR (TITLE-ABS-KEY(“prefer*”))) AND (LIMIT-TO (LANGUAGE, "English”)) AND (LIMIT-TO (DOCTYPE, "ar”))

Because we were interested in nurses’ experience and preferences around shift patterns from their perspective, we only included papers that contained explicit comments or views as reported by nursing staff, whilst excluding papers that made indirect inferences. Papers that were not specific to nursing, as well as news articles and opinions were excluded from the scoping review.

**Fig 1:**
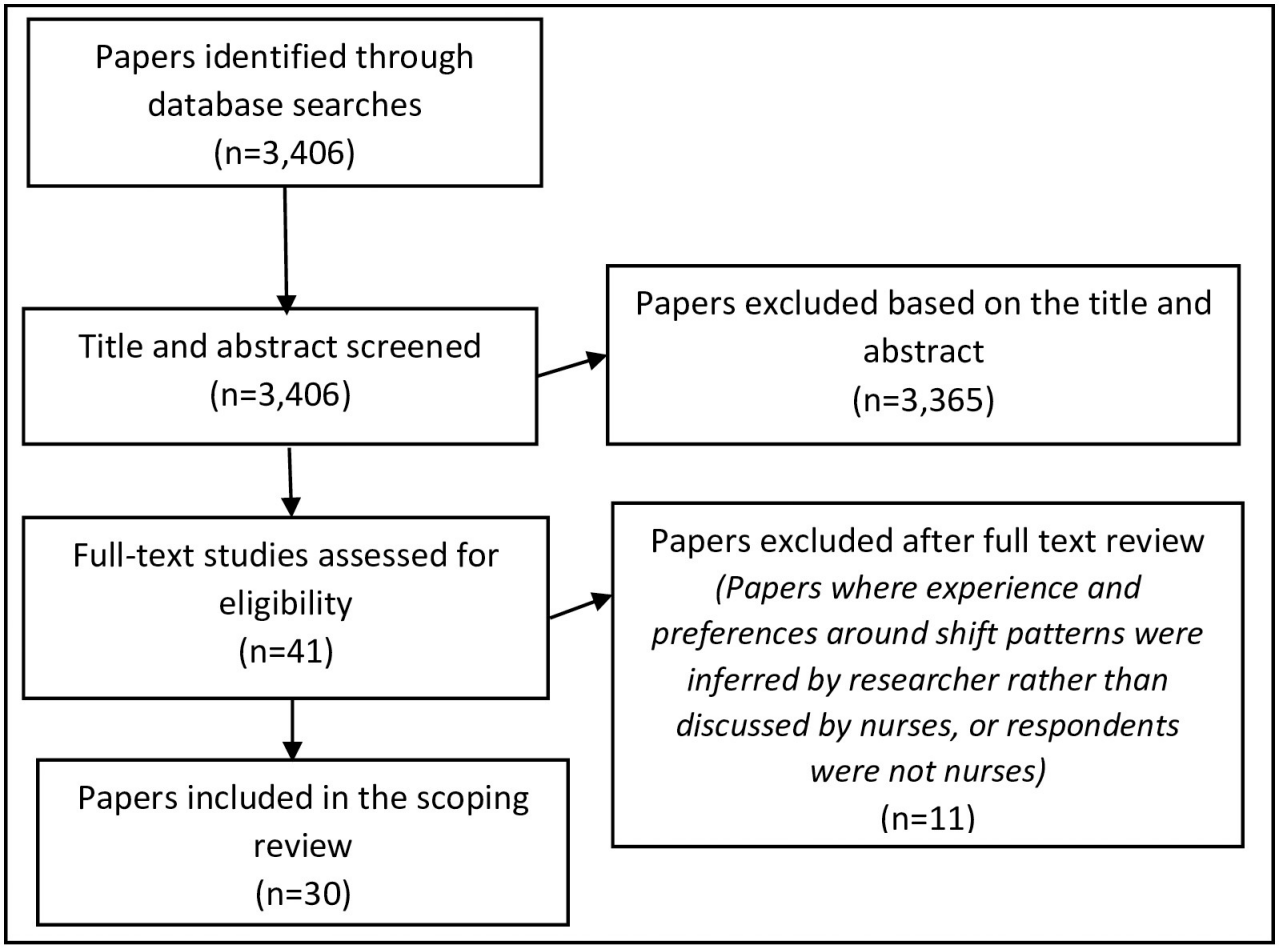
Prisma flow diagram of the literature search and screening process for the scoping review on nurses’ experience and preferences around shift patterns.

OE applied the inclusion/exclusion criteria to screen all titles and abstracts, after which CDO and PG reviewed the selections. All authors agreed that the sample of articles selected for full-text review were relevant for the research question. OE extracted data from relevant studies and met regularly with CDO and PE to discuss findings. It was during these meetings that the key concepts were discussed and refined. Any uncertainties about inclusions and exclusions were also discussed between OE, CDO and PG. The Prisma flow diagram describes the literature search and screening process in details (Fig 1).

## Analysis

To understand the landscape of the literature, we extracted publication date, respondents’ roles (i.e. registered vs unregistered nurse), working place and shift characteristics for each study. Using Excel^®^, we tabulated a spreadsheet where results were recorded and organised by themes based on the authors’ findings. Using an iteration process by re-reading and analysing our results, we separated results based on the advantages and disadvantages of various shift patterns. Results reported on three core aspects of shift work, namely shift work *per se*, shift length and time of shift.

## Results

Firstly, we provide a description of our literature findings based on the number of articles published by date, geographic setting, clinical setting, research methodology and type/length of shift. Secondly, we use a narrative to synthetize the results of the scoping review based on the three aspects of shift work that emerged from the review.

### Numerical analyses of included studies

Three thousand four hundred and six (N = 3,406) records were retrieved from the database searches (Fig 1). 30 papers published between 1993 and 2021 were judged eligible. Half of the papers (n = 16) were published between 2018 and 2021. The largest group of papers came from the USA (n = 8). The UK (n = 7) and Australia (n = 6) had the second highest output. Other papers came from Asia (Iran, n = 2, China, n = 1, India, n = 1 and Cambodia, n = 1), other European countries (one each from Germany, Norway and Sweden respectively) and Canada (n = 1).

Most studies were undertaken with staff working in Acute Hospital wards (n = 27), 2 studies took place in Acute Mental Health Trusts and one study used staff from multiple settings (i.e. Acute Hospitals, Community Trusts and Care Home). The majority of papers included only registered nurses (RNs) (n = 23), whilst other papers included other nursing staff (healthcare assistant and other staff, n = 2, nursing assistant, n = 2, midwives, n = 1 and student nurse, n = 1). One paper focussed solely on unregistered healthcare staff (n = 1). The number of participants ranged between 10 [24] and 1355 [25], with female participants representing the majority of the sample.

Fifteen papers used semi-structured interviews. Four papers used questionnaire with open-ended questions and another four papers used qualitative interviews. Two papers used mixed-methods with a qualitative component and five papers used focus groups. Each paper included a discussion about nurses’ experiences and preferences around shift work from their perspective.

### Overview of themes

The studies we found reported on three aspects of shift work: namely (i) shift work *per se* (e.g. the mere fact of working shift without referring to its length or time), (ii) shift length, and (iii) time of shift, referring to *when* shifts were occurring (i.e. morning, evening, or night); and, in some cases, whether these shifts were worked as part of a rotating or fixed schedule.

Discussions on shift work *per se* were never the focus of a whole study. Rather, nurses were in certain case describing the impact of shift work on their work or life without referring to the length or time. This concerns more than half of the studies (n = 18). When we consider the length of shift, studies focussing on long (12+ hour) shifts were more common (n = 8, including one study investigating 24-hour shifts, Koy, Yunibhand [26]). Seven studies compared 12 hour shifts with other lengths of shift, such as 4, 8 or 10 hours. In these studies, discussion and results were mainly focussed on 12 hours shifts, however. Studies focussing specifically on nurses working short shifts were less common (n = 2).

The third aspect of shift work describes shifts based on their occurrence during the day (n = 11), including studies with night shifts as sole focus (n = 9). The remaining studies (n = 4) described shifts as follows. Epstein, Söderström [27] used a basic shift description, such as ‘morning and evening’ or ‘morning, evening and night’. Gifkins, Loudoun [28] referred to nursing schedule as shift including ‘late night’ or ‘overnight work’. de Cordova, Phibbs [29] used the terminology off-shift (night and weekend), whilst Bauer [30] simply mentioned ‘early shift’ (6 to 7 am start) without indicating the overall number of hours of the shift worked. Table 2 provides a summary of all studies included in the scoping review.

**Table 2:** Papers included in the scoping review (arranged by aspect of shift and descending chronological order).

Shift aspect & patterns	Author(s) and date	Country of origin	Role	Setting	Sample size	Aims of study	Methods	Theme(s)	Aspect of shift(s)
**Shift length: 24 hours**	Koy, V., et al. (2020)	Cambodia	RNs	Hospital	30	To explore the perception and experiences of 30 ICU RNs regarding their working 24h shifts	Focus groups	Exhaustion Compromised hospital care	Shift work *per se* Shift length
					Female = 17				
					Male = 13				
**Shift length: 12 hours**	Suter, J. and T. Kowalski (2020)	England	RNs and HCAs	Acute mental health trust	70	To examine the impact of extended shifts on employee strain in a large mental healthcare organisation	Semi-structured interviews	Context (challenge related to patient care) Consultation process Modification of context with introduction of extended shifts Impact of modified context on strain Temporal strain-based spillover	Shift work *per se* Shift length
					Female = 54				
					Male = 16				
**Shift length: 12 hours**	Suter, J., et al. (2020)	England	RNs, HCA & managers	Acute mental health trust	70	To evaluate how employees in acute mental health settings adapt and respond to a new 12h shift system from a wellbeing perspective	Semi-structured interviews	Perception of quality of care (improvement and deterioration) Stamina and pacing of work to complete shift Social support and reflection Significant factors influencing 12-hours shift: old age Significant factors influencing 12-hours shift: public healthcare commitment Significant factors influencing 12-hours shift: work life balance	Shift length
					Female = 54				
					Male = 16				
**Shift length: 12 hours**	Ose, S. O., et al. (2019)	Norway	RNs	Hospital	24	To record the experiences of 24 nurses working 12h shifts	Semi-structured interviews	Organisational shift structure Health consequences Quality of patient care Family situation and friends Tasks at work	Shift work *per se* Shift length
					Female = 24				
**Shift length: 12 hours**	Webster, J., et al. (2019)	Australia	RNs	Hospital	266	To investigate the effect on nurses and patients of 8h rostering compared with 12h rostering among 266 RNs	Questionnaire with open-ended questions	Patient care Increase in professional development Improvement in communication with all levels of senior staff Health and hygiene Social and leisure Recruitment and retention	Shift length
					Female = 209				
					Male = 57				
**Shift length: 12 hours**	Parkinson, J., et al. (2018)	USA	RNs	Hospital	30	To explore the perceptions of rehabilitation nurses who are working in or who have worked 12h shifts in an acute rehabilitation hospital and to identify the advantages and disadvantages of 12h shifts	Mixed-method study with qualitative questions	Increased time off-work Continuity of care Increased fatigue Changing patient assignments	Shift length
					Female = 27				
					Male = 3				
**Shift length: 12 hours**	Thomson, L., et al. (2017)	England	Unregistered healthcare staff	Hospital, Community trusts, Care homes	25	To explore unregistered healthcare staff’s perceptions of 12h shifts on work performance and patient care	Focus groups	Nurses perceived that 12-hour shifts would benefits patients through continuity of care Satisfaction with performance through continuity of care Negative impact of staff fatigue Other factors moderate the impact of 12-hour shifts	Shift work *per se* Shift length
					Female = nr				
					Male = nr				
**Shift length: 12 hours**	McGettrick, K. S. and M. A. O’Neill (2006)	Scotland	RNs	Hospital	54	To elicit critical care nurses’ perceptions of working 12h shifts	Focus group	Preferences for 12-hour shift Most popular "Strongly agree": Patient care perceived to be improved; Job satisfaction; Off duty; Family life Most popular "Disagree": Communication; Fatigue/freshness, Education Most popular "Not sure": Education, Communication Staffing levels Work breaks	Shift work *per se* Shift length
					Female = 50				
					Male = 4				
**Shift length: 4 or 8, 8 and 12**	Gao, X., et al. (2020)	China	RNs	Hospital	14	To explore 14 nurses’ experiences regarding shift patterns while providing front-line care for COVID-19 patients in isolation wards of hospitals in Shanghai and Wuhan	Semi-structured interviews	To assess the competency of nurses to assign nursing work scientifically and reasonably To reorganise nursing workflow to optimise shift patterns To communicate between managers and front-line nurses to humanise shift patterns Nurses’ various feelings and views on shift patterns	Shift work *per s*e Shift length
					Female = 12				
					Male = 1				
**Shift length: 8, 10 and 12 hours**	Haller, T., et al. (2020)	USA	RNs	Hospital	190	To explore clinical nurses’ perspectives of shift length among 190 clinical nurses	Questionnaire with open-ended questions	Preferences for 12-hour shift Improved work-life balance Improved patient care Burnout & reduction in physical and mental health	Shift length
					Female = nr				
					Male = nr				
**Shift length: 10 and 12 hours**	Horton Dias, C. and R. M. Dawson (2020)	USA	RNs	Hospital	21	To explore hospital shift nurses’ experiences and perceptions of influences on making healthy nutritional choices while at work	Semi-structure interviews	Nursing roles and responsibilities restrict freedom of movement and minimize individual control over dietary practices Hospital food environment is oppressively unhealthy Free food is currency and influences consumption Shift work is a major barrier to healthy eating	Shift length
					Female = nr				
					Male = nr				
**Shift length: 8 and 12 hours**	Baillie, L. and N. Thomas (2019)	England	RNs and NAs	Hospital	22	To investigate how nursing care is organised on wards where nursing staff work different lengths of day shifts, and how length of day shift affects the staffing of wards	Qualitative interviews	Organising nursing care and staff activities Staffing wards with different length of day shift	Shift work *per se* Shift length
					Female = nr				
					Male = nr				
**Shift length: 8 and 12 hours**	Haller, T. M., et al. (2018)	USA	RNs	Hospital	87	To explore clinical nurses’ perceptions of 12h shifts versus traditional 8h shifts	Semi-structured interviews	Perception of relationship of shift length to patient outcomes Flow of the workday Home wellness Physical toll of shift length	Shift work *per se* Shift length
					Female = nr				
					Male = nr				
**Shift length: 8 and 10 hours**	Centofanti, S., et al. (2018)	Australia	RNs and midwives	Hospital	22	To investigate the way nurses and midwives utilised napping and caffeine countermeasures to cope with shift work, and associated sleep, physical health, and psychological health outcomes among 130 shift-working nurses and midwives	Qualitative interviews	Napping Caffeine	Shift work *per se* Shift length
					Female = 19				
					Male = 3				
**Shift length: 8 and 12 hours**	Baillie, L. and N. Thomas (2017)	England	RNs and NAs	Hospital	22	To explore how length of day shift affects patient care and quality of communication between nursing staff and patients/families in older people’s wards	Mixed-method study with qualitative questions	Effects of day shift length on patient care Effects of day shift length on continuity of care and relationships Effects of day shift length on communication with patients and families	Shift work *per se* Shift length
					Female = nr				
					Male = nr				
**Shift length: 8 hours**	Rathore, H., et al. (2012)	India	RNs	Hospital	60	To have an insight into the problems faced by female nurses in shift work	Qualitative interviews	Lack of sleep Physiological (reduced alertness) and psychological fatigue	Shift work *per se* Shift length
					Female = 60				
**Shift length: 8 and 12 hours**	Reid, N., et al. (1994)	England	Student nurse	Hospital	47	To report on the attitudes of nurse educators and students to the 12-hour shift and their views on the impact such a shift has on nursing education among students registered general/mental nurse	Qualitative interviews	Preference for 12-hour shift No change in quality and continuity of care Leisure Recruitment Study time	Shift length
					Female = 46				
					Male = 1				
**Time of shift: Night shift**	Landis, T. T., et al. (2021)	USA	RNs	Hospital	16	To describe and interpret the lived experience of hospital night shift nurses taking breaks and the meaning of this phenomenon as it relates to the workplace.	Semi-strucured interviews	Primary purpose of breaks: Eating Ability to take breaks depended on unit-level structures Breaks were perceived as a luxury	Time of shift
					Female = 14				
					Male = 2				
**Tine of shift: Morning, evening and night**	Epstein, M., et al. (2020)	Sweden	RNs	Hospital	11	To explore newly graduated nurses’ strategies for, and experiences of, sleep problems and fatigue when starting shift-work	Semi-structured interviews	Factors contributing to sleep problems Strategies for sleep Experiences of fatigue Strategies for fatigue	Shift work *per se* Time of shift
					Female = 10				
					Male = 1				
**Time of shift: Night shift**	Smith, A., et al. (2020)	USA	RNs	Hospital	39	To elicit night shift nurses’ perceptions of drowsy driving, countermeasures, and educational and technological interventions.	Semi-structured interviews	Drowsy driving experience Existing countermeasures Social influences Barriers Educational program perspectives Drowsy driving mitigation technology perspectives	Time of shift
					Female = 26				
					Male = 13				
**Time of shift: Night shift**	Matheson, A., et al. (2019)	Australia	RNs	Hospital	10	To explore women’s experiences of working shift work in nursing whilst caring for children	Semi-structured interviews	Being guilty Being juggler	Shift work *per se* Time of shift
					Female = 10				
**Time of shift: Night shift**	Books, C., et al. (2017)	USA	RNs	Hospital	101	To study night shift work and its health effects on nurses	Questionnaire with open-ended questions	Health promotion Night shift effect on health Health perception	Shift work *per se* Time of shift
					Female = 88				
					Male = 13				
**Time of shift: Shifts including late night and overnight work**	Gifkins, J., et al. (2017)	Australia	RNs	Hospital	21	To compare perceptions of nurses exposed to short- or longer-term shift work and their experiences working under this type of scheduling	Semi-structured interviews	Working in shifts Coping with shifts Support from family, friends and senior nurses	Shift work *per se* Time of shift
					Female = 21				
**Time of shift: Night shift**	West, S., et al. (2016)	Australia	RNs	Hospital	1355	To develop a conceptual model of nurse-identified effects of night work among 1355-night working RNs employed in a state/public health system	Questionnaire with open-ended questions	‘Lives’ of night working nurses ’Bodies’ of night working nurses ‘Work’ of nurses at night Nurses’ workplace at night	Shift work *per se* Time of shift
					Female = 115				
					Male = 192				
**Time of shift: Off-shift (night and weekend)**	de Cordova, P. B., et al. (2013)	USA	RNs	Hospital	23	To qualitatively explore 23 RNs perceptions of off-shift nursing care and quality compared with regular hours	Semi-structured interviews	Collaboration among self-reliant night nurses & teamwork Completing more tasks Taking a breather on weekend day shift New nurse requirement to work at night first before working during the day Mixture of registered nurse personnel Night nurse perception of under-appreciation	Time of shift
					Female = 20				
					Male = 3				
**Time of shift: Night shift**	Faseleh Jahromi, M., et al. (2013)	Iran	RNs	Hospital	20	To describe 20 Iran novice nurses’ perception of working night shifts	Focus groups	Value system Physical and psychological problems Social relationships Organizational problems Appropriate opportunity	Time of shift
					Female = nr				
					Male = nr				
**Time of shift: Night shift**	Powell, I. (2013)	Australia	RNs	Hospital	14	To report a study that explored the experiences of night-shift among 14 nurses, focusing on employee interrelationships and work satisfaction.	Semi-structured interviews	Work relationship Work environment Work practices	Time of shift
					Female = 14				
**Time of shift: Night shift**	Fallis, W. M., et al. (2011)	Canada	RNs	Hospital	13	To explore nurses’ perceptions, experiences, barriers, and safety issues related to napping/not napping during night shift	Focus groups	Environmental scan Impact of napping: energized or disoriented Consequences of not napping: foggy thinking Mixed views about management and the public regarding nurses napping during night shift	Shift work *per se* Time of shift
					Female = 11				
					Male = 2				
**Time of shift: Night shift**	Nasrabadi, A. N., et al. (2009)	Iran	RNs	Hospital	18**	To describe the perceptions held by Iranian registered nurses (IRNs) concerning their night shift work experiences	Semi-structured interviews	Socio-cultural impacts of night work Health-related impacts of night work Night work as an opportunity for gaining more clinical experiences and learning more	Shift work *per se* Time of shift
					Female = 11				
					Male = 5				
**Time of shift: Early shift (6 to 7am)**	Bauer, I. (1993)	Germany	RNs	Hospital	14	To explore perception of German nurses of early shift	Semi-structured interviews	The impact of rising early on the individual The detrimental effect of alternating shifts on well-being Disturbances to personal life The rationale for early start Consequences for patients	Time of shift
					Female = 13				
					Male = 1				

HCA = Health Care Assistant NA = Nursing Assistant nr = not reported

**As reported by the authors

Findings from the review are summarised and presented according to these three aspects of shift work, namely shift work *per se*, shift length, and time of shift. Within each aspect of shift work the impacts of shift pattern on experience and preferences are discussed.

### Shift work *per se*

An aspect discussed by nurses when reflecting on shift patterns was shift work *per se*, meaning the mere fact of working shifts as opposed to a regular “9 to 5” day job.

Nurses often reported detrimental mental and physical health as a result of working shifts, as well as failure to adopt healthy strategies, such as walking or exercising when working shifts [31]. Working shifts could also act as a barrier to healthy eating with nurses reporting unhealthy eating practices [32, 33], such as overconsumption of caffeine and energy drinks, resulting in poor sleep and health outcomes [34]. Such habits could stem from different sources, including exhaustion and job-related stress which further exacerbated circadian rhythm disruption [33]. Notwithstanding the negative effect of shift work on nurses’ health and wellbeing, presenteeism (i.e. going to work when being sick resulting in lower engagement and productivity) was often brought up, in particular by nurses who are mothers. They reported feeling guilty about calling in sick, because staff shortages would mean that their shift would have to be covered by a colleague forced to do overtime [24]. This was amplified by nurses experiencing increased workload as a result of staff shortages during their shifts [25, 26, 35, 36].

Nurses asserted that working shifts could in certain cases not be conducive for rest and napping [34] or restricting access to planned break periods [37–39]. Fatigue was often discussed in relation to shift work, and it was found to be exacerbated by working more than two consecutive long shifts [37, 40]. Noticeably, nurses observed that the reduction in consecutive shifts decreased work absence [38]. Fatigue could be manifested by physical pain [41], difficulty to transition from day to night-time (and vice-versa) [42], reduced concentration [27, 37], difficulty in taking decisions or emotional manifestation, such as being easily annoyed or unengaged during shift [27].

One study revealed that preference for shift work varied depending on the length of time (i.e. experience) in working shift [28]. Experienced nurses (i.e. at least three years of shift work experience) had a preference for shift work because it aided in their work-life balance. They benefited from the support of their family, friends and senior nurses which positively impacted on their domestic and children caring responsibilities. In contrast, inexperienced nurses reported being isolated and missing out on family and social activities, because friends (more often than family) did not always understand the time constraints of working shifts [28]. The ability to request for their own roster also helped nurses to cope with shift work [28]. Relatedly, nurses choosing their own roster were more satisfied with their job [43]. In contrast, nurses were more reluctant to accept, adapt or prefer a particular shift pattern when it was mandatorily imposed [26, 37, 44].

One study by Gao et al. [35] offered a perspective on how the experience of shift work was affected by the COVID-19 pandemic. As a result of the increase in patient numbers and their acuity, and, consequently in workload, nurses highlighted the need to adjust shift patterns dynamically according to the workload. They also emphasised the importance to account for nurses’ knowledge, skills and abilities during shift scheduling, as well as considering their physical and mental experience [35]. Based on their shift experiences during the COVID-19 pandemic, nurses asserted that their perspectives should be taken into consideration to humanise shift patterns. Besides, communication between managers and front-line nurses should be strengthened to understand nurse perspective when scheduling shift schedules [35].

### Shift length

Nurses reflected on their experience and preferences around the length of shifts. Our review report on two main lengths of shift, short shifts (<12 hours), including 4-hours [35], 8- or 10-hour shifts; and long shifts (≥ 12 hours and more), including one study focussing on 24-hour shifts [26].

A factor discussed by nurses in relation to different shift lengths was handovers. Studies consistently highlighted how nurses believe the additional handovers resulting from shorter shifts posed a threat to safety in terms of miscommunication of patient information. One study found that nurses were concerned about the additional handover when moving from long to short shifts, such that the information did not always reach the next shifts [36]. Nurses believed the introduction of an additional handover as a result of moving to 8 hour shifts had led to a higher risk of information being miscommunicated, similar to a Chinese Whispers effect [36]. When the length of shifts was reduced from 8 to 4-hour during the COVID-19 pandemic, nurses described how they felt shorter shifts had led to more handover errors [35]. In another study, nurses reported that communication with all levels of senior staff improved, probably as a result of the extra time that long shifts offered [45]. However, not all studies concluded that long shifts were beneficial to improve communication. One study mentioned that implementing long shifts led to a disruption in communication with colleagues [38].

The loss of one handover when implementing 12 hour shifts limited opportunities for clinical education [38]. It also led to a decrease in informal social support, reduced opportunities for sharing good practices and reflection time. Nurses reported being more isolated, because core staff increasingly worked alongside agency staff which worsened collegiality [46]. In contrast, in one study, nurses reported an increased access to professional development education after 12 hour shifts were introduced. The higher rate of professional development leave was supported by organisational data [45]. One study highlighted how the ability to study was affected by the subsequent tiredness after working long shifts [47]. In some instances, the introduction of 12 hour shifts also reduced nurse confidence in their clinical skills and knowledge following extended time away from a dynamic ward environment [44, 46]. Nurses working short shifts also declared having limited access to education, teaching or staff development as a result of work intensity [36].

Nurses believed there had been an increase in staff turnover after 8 hour shifts were imposed, because staff did not like or did not adapt to the new shift pattern [36]. Some nurses feared 12 hour shifts would cause recruitment challenges of adequately trained nurses [47]. In contrast, another study reported that nurses believed 12 hour shifts would improve retention of experienced staff due to the shift flexibility and ability to increase nurses’ morale, possibly because those nurses reported a strong preference for this shift pattern [45]. Relatedly, some nurses believed the implementation of long shifts had improved staffing levels, with more nurses available during the shifts [38].

Across different studies there were contrasting results for nurses’ views about the impact of long shifts on the quality and continuity of care after 12 hour shifts were implemented. Long shifts were perceived by unregistered healthcare staff [37] and nurses to improve patient and continuity of care [38, 41, 42, 45, 46, 48, 49]. The implementation of long shifts rendered possible the full completion of their nursing tasks, as evidenced by fewer interruptions of work tasks and the possibility to focus on their tasks for longer [41, 42]. Nurses perceived improved communication with patients [42], as they were able to care for the same patients throughout their shifts [48]. They also reported achieving more nursing care with their patients resulting from the extra time long shifts offered [37, 41, 42].

However, in a few studies nurses reported a deterioration in the quality of care they were delivering in the last (four) hours of the shifts [47]. Nurses also had mixed views about the effect of short or long shifts on quality of care, stating their uncertainty about which shifts improved patient care [36, 40]. Nurses reported that the introduction of 12 hour shifts amplified their job strain and left some nursing tasks incomplete because of the intensity of work over an extended period of time [46]. Another study revealed that frequent changes in assignments during long shifts could negatively impact continuity of care, as nurses could not complete their nursing care with the same patients throughout their shifts [48]. The perceived increase in continuity of care over time, in the control over nursing tasks completion, and in the improved communication with patients as reasons for preferring 12 hour shifts [37, 42].

Fatigue (or tiredness) was a feature reported by nurses across all shift lengths, suggesting nurse endure a physical burden when working shifts irrespective of their length. Nursing staff working short [37, 43] and long shifts [27, 38, 40–42, 44, 47–49] reported experiencing fatigue when working shifts. Nurses reported having to pace themselves to complete their shifts, reflecting the necessity for increased physical, mental and emotional stamina when working long shifts [44, 46]. Exhaustion during and after working long shifts was also a common feature reported by nurses [26, 33, 44]. They described that working long shifts led to burnout, reduction in physical and mental health [49].

Nurses’ sleep patterns were reported to be negatively affected by both short [43] and long shifts alike [27, 33], apart for one study where nurses reported an increase in their sleep hours after moving to 12 hour shifts [45]. Some nurses working 12 hour shifts reported fatigue was more manageable since they benefited from more days off-work to recover [38, 45, 47]. In one study, some nurses recognised that fatigue was an adverse effect of long shifts, but they found this was mitigated by the increased number of days off-work [46]. The extra days off and improved work-life balance were often mentioned as the reason for a preference for long shifts [36, 38, 41, 42, 44–49]. Work-life balance was also positively rated by nurses working 8 hour shifts [49]. Noticeably, one study reported that nurses’ views of shift length on their leisure was mixed. The contrast may stem from the fact that the population of interest were student nurses, whose educational commitments may restrict their leisure time [47].

Long shifts was associated with anticipated anxiety to return to work, where nurses apprehended returning to a challenging and unpredictable workplace [44]. Stress could also stem from the changes in skill mix resulting from the implementation of 12 hour shifts. For instance, nurses’ supervision increased as a result of using extensive agency and bank staff to cover for sickness absence. Indeed, these temporary staff were unfamiliar with the ward and needed more support from substantive staff [44]. In contrast, in one study nurses felt that working long shifts reduced sickness and/or family leave, as nurses could benefit from extra days off work [45]. Nurses also reported being able to take more planned breaks when working long shifts [41, 42], albeit this was dependent on patient acuity and case mix [38].

Whilst two studies revealed nurses were satisfied or had a preference for short shifts [42, 49], more studies showed nurses preferring long shifts [37, 38, 41, 45, 47, 49]. Acceptance of 12 hour shifts was facilitated when staff were consulted prior to moving to long shifts, and, in some cases, when the request to change to 12 hour shifts came from staff themselves [41, 45, 47]. Individual characteristics and personal circumstances (e.g. age, marital status, grade or presence of children) influenced the extent to which nurses could adapt to the new shift patterns. For instance, the strain of long shifts on wellbeing was particularly intense for older nurses [25, 44, 46]. A further predictor of adaptation to 12 hour shifts was public healthcare commitment: when nursing staff were devoted to the National Health Service (NHS) and their Trust, nurses expressed they accepted the move to 12 hour shifts because they wanted to be helpful towards a struggling sector and employer [46]. Furthermore, nurses reported wanting to be seen to be coping with long shifts as a means of improving their team’s morale [46]. Nurses also reported that childcare costs were reduced when working long shifts as they allowed them to spend more days at home caring for their children [42].

### Time of shift

Nurses working night shifts described how the lack of resources, for example other healthcare professionals and administrative staff, led to higher collaboration among them, higher sense of cohesion and better teamwork [25, 29], but also led to difficulty to taking their breaks [50]. Nurses working night shifts experienced increased autonomy and fewer interruptions to planned healthcare as a result of fewer family visits [25, 51]. More specifically, working night shifts allowed nurses to carry out indirect (e.g. administrative) as well as direct clinical patient care with more autonomy [25]. Relatedly, night working enabled nurses to feel more independent and more skilled, fostering their desire to continue working at night [32, 52].

However, lack of resources and of support from other professionals during the night shift led to significant concerns for nursing staff, who reported feeling not considered and appreciated by staff on day shifts [51]. Communication problems between day and night shift nurses could occur, where night nurses felt disconnected from and neglected by day nurses, and struggled to access relevant (patient) information [25]. They perceived their role as under-appreciated by other nurses [29], referring to a universal consensus that night shift nurses are perceived to be practising less or not all [51]. Some nurses experienced fear and insecurity in the last hours of their night shifts resulting from lack of resources [52], exacerbating worries around patient and professional safety [25]. Other nurses denounced inferior working conditions in comparison to their daytime counterparts, including a perception of minimal leadership [51], as well as a lack of welfare facilities [52]. Staffing levels and skill mix were also discussed in relation to night work; night shifts were often covered by temporary staff, and substantive staff felt this was a positive thing because staffing levels could be maintained [29].

Nurses working night shifts valued patient care, as evidenced by the importance they placed in knowing their patients’ conditions and care needs [51]. Nurses’ sense of duties and responsibilities firstly towards their patients also meant they could neglect taking their breaks [50]. The more ‘relaxed’ pace during weekend day shifts allowed nurses to focus more on their patients [29]. However, nurses reported that staffing levels were often inadequate at night [25] or workload was too high [51]; leading staff to report that the quality of care they delivered was negatively affected. Inexperienced nurses (less than one year nursing experience) perceived increased work pressure when working night shifts [52]. Relatedly, nurses experienced tremendous workload when working early shifts, which rendered the work unappealing altogether [30]. Nurses working off-shifts (night and weekend) also revealed they completed more tasks as a result of inadequate staffing level during these shifts [29].

Nights shifts gave rise to educational and clinical learning opportunities from which nurses benefited [25, 32, 52]. Nurses stated that night shifts were more relaxed and some e-learning not available during the day were available at night [25]. In contrast, nurses also reflected on the conundrum of night shifts where their learning opportunities were perceived as suboptimal. This was evidenced by a reduced or lack of education access in comparison to their daytime counterparts, despite added responsibility [51].

Depression, tiredness [31] and fatigue were experienced by nurses working night [25, 51, 52] and day (including evening) [27] shifts. Nurses working night shifts also experienced drowsy driving after completing their shifts [53]. Nurses adopted unhealthy strategies to combat fatigue and adapt to late or night shifts. This included caffeinated drinks and snacking on sugary foods, as well as drinking alcohol to rest and recover after night shifts [27, 28, 39]. Most common strategies to combat drowsy driving after night shifts included listening to music, talking on the phone with relatives, as well as unhealthy snacking [53]. Nurses working early shift also reported being exhausted, as evidenced by their physiological system not being used to start working so early [30].

Nurses asserted their sleep patterns were negatively affected by both early [30] and night shifts [25, 27, 31, 32, 52]. Sleep deprivation stemmed from nurses’ inability to rest between late to early shifts [30], insomnia [52], feeling sleep-deprived while working or the difficulty to achieve a normal sleep pattern after the completion of night work [25]. Nurses reported suffering from anxiety, nutritional imbalance, changed physical appearance and skin pigmentation as a result of working night shifts [32]. Early shifts also caused anticipated anxiety to return to work, with nurses finding difficult to disconnect from work and over-processing their nursing tasks while at home [30]. Stress could also results from dysfunctional organisational structures or poor workplace conditions when nurses worked night shifts [52].

Nurses worked a variety of shift work schedules (i.e. permanent or rotating) and preferences for shift patterns could vary depending on the shift work schedules they were assigned to. For instance, there is evidence nurses preferred working permanent night shifts in comparison to rotating shifts [24, 25, 31]. And there is also evidence that nurses working night shifts on a permanent basis were satisfied with this shift pattern [29]. Whilst working night shifts was exhausting, it also gave nurses a sense of fulfilment [32]. Nurses stated that the environment was more relaxed at night, as evidenced by reduced noise, fewer nursing task interruptions and increased focus-thinking time.

Nurses were able to care for their family when working late or night shifts, despite financial and non-financial constraints for single-parent families [25], or pervasive fatigue [51]. For instance, nurses working night shifts declared benefiting from shared parental responsibility and their presence at home during the day meant they avoided using after-school care [25]. However, nurses also described their sense of guilt when leaving their children to work night shifts, as well as the difficulty in co-ordinating their family and social life [24]. Working early [30] or night shifts could also result in nurses being or feeling isolated from family and friends due to unsociable working hours [25, 28, 31, 51, 52].

## Discussion

This is, to our knowledge, the first scoping review to provide a comprehensive overview of research on nurses’ experiences and preferences around shift patterns in the nursing literature. A broad range of international studies were found and included, reflecting the global interest in understanding nurses’ perspectives when reflecting on the impact of shift patterns. Findings mostly focussed on either the mere fact of working shifts, the length of shifts or the time of shift, although issues such as number of days worked in a row and ability to choose shifts also emerged. We found that all different aspects of shift work elicited a variety of positive and negative views from nursing staff, with no single shift pattern described as without limitations. In the same vein, we found that some topics or issues reported by nurses align or contrast with the corresponding quantitative evidence on nursing shifts (Table 3).

**Table 3:** Comparative table between subjective and quantitative evidence around nursing shift patterns.

Theme	Quantitative evidence	Subjective experience
**SHIFT WORK *PER SE***		
Nurses’ health and wellbeing	Negative health outcomes such as cardiovascular diseases, gastrointestinal and metabolic disorders (type 2 diabetes: metabolic syndrome). [54] Sleep disruption and sleepiness after shift work [55] Fatigue when working during time-off [56]	Unhealthy eating and eating at the wrong time [33, 34] Missing breaks during shift work [34] Physiological fatigue resulting from lack of sleep after working shifts [43]
**SHIFT LENGTH**		
Nurses’ health and wellbeing	Fatigue and insufficient/poor-quality sleep when working 12h shifts [4, 57]. Rotas are often designed based primarily on service demands rather than staff needs [58, 59] Higher level of dissatisfaction when working long (12h+) shifts [13, 14, 60, 61]. Higher burnout scores for nurses working 12h shifts [13, 62]. Nurses working 12h shifts were more likely to be satisfied with their job [19], experienced less burnout, and had a reduction of 5.9 points on the emotional exhaustion scale [18] Nurses choose or accept 12h shifts to reduce childcare-related issues [62] Increase in short [58] and long-term sickness absence when working higher proportion of 12h shifts [63, 64]. Reduced sickness absence when implementing participatory working time scheduling [65]	Exhaustion [26, 44], reduction in physical and mental health [49] when working 12h shifts. Fatigue [37, 41, 48] accentuated by anxiety when working extended shifts [44]. Anxiety around returning to work in a challenging and unpredictable environment when working long shifts [44]. Nursing staff would prefer more autonomy, better roster planning and less consecutive shifts to reduce fatigue and tiredness [37]. Job strained amplified when working extended shifts [44]. Intensity of work during 12h shift implementation require nurses to pace themselves [46] Reduced opportunities for informal social support, increase in staff isolation, worsening colleague relationship as a result of 12hr shift implementation, reduced opportunities for sharing good practice with colleagues and less ‘downtime’ to reflect [46] Improvement in work-life balance promoted by consistent schedules when working long shifts, allowing for more family/friend time [38, 41, 49]. Days off-work mitigate adverse effects (such as fatigue) of 12h shifts, satisfaction with compressed week but limitation for social leisure when working 12h shifts [44, 46]. Reduction in childcare related issues when working 12h shifts [42] but difficulties to attend social events [45] Reduction in sick and family leave when working 12h shifts [45]. Fatigue more manageable when working 12h shifts (e.g. enough time to recover) [38, 45]. Satisfaction with [45] or preference for 12h shifts [47, 49] due to increased time off work and time with family. Nurses tend to adapt to 12hr shift after its implementation [44] but older nurses find it difficult [46].
Patient care and workload	More difficult for nurses to stay awake when working long shifts [66], and nurses are more likely to report risking making a medication error compared to nurses working short shifts [66, 67]. More patient missed care during 12h shifts [10, 11] No difference in nurse reported quality of care for nurses working 8 and 12h shifts [18]. 12h shifts were associated with poor quality of care, lower patient safety and missed care [10]. Quality of care, patient safety and patient satisfaction negatively associated with long shifts [14, 61, 68] Nurses working shifts long shifts were less likely to report assignments that foster continuity of care, albeit the association was not significant [15] More care hours per patient per day (and higher costs) during long shifts [12] Working long shifts was associated with reports of patient care information being lost during handovers, although association was not significant [15] Delayed (patient) observations more when higher proportion of the hours worked by healthcare assistants were part of long shifts [16]	Perceived reduction in quality of care: missed care and decrease nursing care quality and patient safety when working long shifts [26, 44]. Respondents reported no medical mistakes after changing from 8 to 12h shifts. Longer shifts contributed to a reduction of nursing tasks’ interruption, more continuity of care [42, 48], better anticipation of patients’ needs [41] and improved communication with patients [40] Difficulty to familiarise with new/revised patient care plans after 12h shift implementation [44]. Assignment frequently changed during 12h shift negatively affecting continuity of care [48] Mixed views on whether 12h shifts had increased the amount of direct nursing care, perceived deterioration of nursing care in the last 4 hours of the day [47] Reduction in handover quality after 12h shift implementation resulting in fewer staff, staff being away for a long period of time, delivered by agency staff [44]
Capacity building	Reduced opportunity for continuing educational programmes, and time to discuss patient care with other nurses when working long shifts [15] Nurses are more likely to report intention to leave when working 12h+ shifts [13, 14, 68]	Increased opportunity for professional development when working 12h shift (in comparison to 8h shift) [45]. Reduced opportunity for educational development because loss of overlap when working 12h shift [38] or difficulty to find time [36] Nurses believed 12h shift would improve retention included an ability to keep experienced staff, have more flexible schedules and increased staff morale. Increase in staff turnover after introduction of 8h shifts: shifts were perceived by nurses as not being flexible [40]
**TIME OF SHIFT**		
Nurses’ health and wellbeing	Night shift associated with disturbed sleep among nurses [5] and higher sickness absences rates compared to day-shift nurses [6]. Working four or more consecutive night shifts in the 28 days preceding sickness absence was associated with an increased likelihood of sickness absence among shift workers [69] Night shift work associated with poorer health, increased absenteeism, higher job dissatisfaction [70] and increased risk of diabetes [71]. Increased level of oxidative stress and anxiety indexes in both day and night shift nurses [72]. Night shift nurses more likely to consume alcohol and smoke [9]	Fatigue after night shifts [52] leading to drowsy driving. Use of caffeine products to stay alert: results of such methods are not conclusive, i.e. not clear whether it helps nurses to stay awake [53]. Lack of sleep and tiredness, sense of isolation resulting from the day-night switch (i.e. nurses being asleep to recover whilst others are awake and active) [25, 30, 31, 51]. Difficulty to transition from day to night shift work [42] Anxiety due to anticipating returning to early shift work [30]. Night shift working nurses noticed physical changes, i.e. change in skin pigmentation and in physical appearance. They are more nervous, eat unhealthily and experience a lack of sleep as a result of working night shifts [32]. New nurses are not dissatisfied with night shifts [29]. Nurses working at night feel they are under-appreciated by day shift nurses (e.g. universal consensus that night shift nurses “don’t do anything”) [29] Sense of guilt when leaving children to work night shifts, as well as the difficulty in co-ordinating family and social life [24]. Ability to care for children during the day but financial constraints for single parents [25]. Ability to attend family and social activities (i.e. during the day) after night shifts, but limited by fatigue [51]. Few or no leisure after working night shifts [52]
Patient care	Night shift workers committed more errors and had decreased performance [73, 74]	Nurses have more autonomy to care for patient during night shifts [25]. Nurses feel patients do not appreciate being woken up during the early shifts (6–7 am) [30] Nurses working night shifts gain more clinical experience and are more autonomous [32]. Nurses value nursing care: they place a great effort in knowing their patients, but the intensity of workload affect the quality of care provided to patients [51]
Workload & capacity building	Opportunities for educational programs during night shifts to promote team building, enhanced communication and interactions among employees [75] Night shift nurses take significantly less 30-min breaks than their day-shift counterpart [76] During night shifts, level of staffing levels in critical care units most likely to be below target [77]	Educational and clinical learning opportunities during night shifts [25, 32, 52] since night shifts are more relaxed and some e-learning not available during the day are available at night [25]. Conundrum of night shifts where learning opportunities are perceived as suboptimal, evidenced by a reduced or lack of education access in comparison to daytime nurses, despite added responsibility [51]. Difficulty to take break: work break not as restful as nurses expected (e.g. ‘barely have time to eat’) [50]. Lack of rest (e.g. napping) during night shifts result in nurses not being efficient (nurses’ cognitive thinking is negatively affected and nurses are irritable) [27, 39] Nurses complete more nursing care because there is less staff [29]. Night shift nurses are supporting each other despite enduring inferior working conditions in comparison to day nurses, including less managerial support, lack of staff and equipment [29], and problems of communication between day and night shifts nurses [51]. Weekend day shifts have a more relaxed pace in comparison to weekday shifts so nurses can better focus on patient care [29]

For example, exhaustion, physical and mental fatigue were outcomes reported by all nurses engaged in working shifts, regardless of their length or time of day. Quantitative research has shown that fatigue increases with the length of shift [57] and is more acute when nurses work during their days off [56]. Inter-shift fatigue (i.e. not feeling recovered from previous shift at the start of the next shift) is also prominent among nurses [4]. In our review, fatigue and exhaustion were recurring outcomes identified across all shift patterns, and they were further exacerbated by high number of consecutive shifts and the COVID-19 pandemic. Notwithstanding the frequent reports of fatigue, nurses did not seem to deploy effective coping systems. For example, studies reported how nurses resorted to unhealthy eating and drinking to survive night and/or long shifts. In addition, a number of studies highlighted how even when fatigued or ill, nurses expressed a sense of guilt towards colleagues and patients and a need to self-sacrifice, which led nurses to work when sick (i.e. presenteeism) and to miss breaks during their shifts.

This feeling of obligation towards colleagues, patients and the healthcare system is well reflected in recent studies [78] and it exemplifies the “Supernurse” culture [79], according to which nurses feel they need to sacrifice themselves, their health or their children’s health [24] for the greater good (i.e. the nursing team and/or their patients). This is a major barrier to nurses taking their breaks, calling in sick when needed and, consequently, to reducing their fatigue levels. This might be further exacerbated by the fact that the majority of nurses are women, including mothers, who feel pressured to juggle their work and childcare responsibilities [80]. Whilst fatigue was often mentioned by nurses, there was little description of their experience of fatigue. Capturing fatigue levels and its manifestation from a subjective perspective is essential, and any interventions that modify shift patterns should consider how fatigue could be impacted from the nurses’ perspective. Having enough days off to recover from shift work-related fatigue was noted in several studies. Changes and adaptations to shift patterns should consider the sequencing of days off between shifts and the cumulative number of days worked and not simply the total days off within the (arbitrary) seven day week.

We found that personal characteristics including age, length of experience, and caring responsibilities affected the experience and preferences around different shift patterns. While these were often mentioned by nurses, there was no discussion on how such personal characteristics represented a constraint for nurses when choosing or working shifts. Further research may explore the extent to which personal circumstances are constraining nurses when choosing or working shifts. Especially considering the evidence that personal circumstances are not consistently taken into account by health services when designing rotas, which are often designed based primarily on service demands rather than staff needs [46, 58, 59].

The majority of the studies included in this review were conducted in Acute Care Hospitals, indicating there is a dearth of investigation of nurses’ experiences and preferences around shift patterns in other settings. These include community and mental health hospitals. Research conducted in these areas is needed, as experiences of nurses working in those settings may differ significantly from those in acute care hospitals.

The perceived impact of shift patterns on patient care and capacity building was inconclusive, with nurses stating both negative and positive views. Some nurses reported higher continuity of care, and a lower risk of information being miscommunicated when working long shifts. In contrast, nurses in some studies felt that continuity was decreased because they were away from the ward for longer due to having more days off. Other studies found no evidence that nurses working 12 hour shifts reported improved continuity of care and less miscommunication of information compared to nurses working shorter shifts [15]. Similarly, there were contrasting views when it came to educational opportunities and shift length. Yet, an observational study found that nurses working 8 hour shifts reported having more education opportunities in comparison to those 12 hour shifts [15]. Large observational studies also show that nurses working 12 hour shifts are less likely to report high quality care or improving safety on their wards compared to those working shorter shifts [10, 68].

We found that nurses tend to prefer shift patterns when they were involved in designing those shift patterns, or, even more, when changes had been adopted based on requests from staff. Two studies captured nurses’ preferences for long shifts using a longitudinal design, enabling to elicit nurses’ preferences before, during and after the implementation of 12 hour shifts [41, 44]. Results confirmed that the mandatory imposition of changes in shift patterns [44] in contrast to a voluntary approach [41] increased the likelihood of nurses disapproval. Having choice and flexibility around shift patterns is a known predictor of increased wellbeing and health [65, 81]. Hence, interventions aimed at modifying shift patterns should consider involving nursing staff to maintain a certain degree of choice.

Our findings that many nurses prefer long shifts and believe working long shifts does not affect patient care largely contradicts the quantitative evidence, where those working long shifts are likely to report lower quality of care and job satisfaction and higher levels of burnout [10, 13, 14, 68]. Higher rates of sickness absence have also been reported for nurses working 12 hour shifts [63]. This contrast between qualitative and quantitative evidence needs further exploring; specifically, further research is required to better understand the mechanisms that lead nurses to prefer some shifts, for example whether a higher degree of choice around shift patterns is a consistent moderating factor for the negative outcomes of long shifts for either staff or patients. Previous research does not seem to indicate this [82], but a deeper investigation including triangulation of roster data and qualitative reports might shed more light.

The evidence from large observational studies does not directly link individual preferences for shifts with patient and care outcomes [62, 83], but it could be that nurses exercise choice for shift patterns that lead to less favourable working conditions because of external considerations, such as childcare. The need to accommodate responsibilities such as childcare might explain why nurses could sacrifice job satisfaction in order to balance demands. This could explain some of the apparent contradiction between these two bodies of research, such that nurses could prefer particular shifts despite the association with occupational burnout and decreased job satisfaction. Indeed, the staff themselves may not make the direct attribution even if their shift patterns played a causal role.

Whilst the scoping review has shed some light on nurses’ experiences and preferences around shift patterns, our study is not without limits. Following the scoping review process, we did not conduct a systematic appraisal of the qualities of studies [22]. Whilst this approach widens the scope of studies included in the review, it may also bias the conclusion of our findings as the strength of the evidence is not being assessed. Noticeably, we also found that most studies come from the USA, UK and Australia, and these findings might not apply to nurses working in other parts of the world. As expected in qualitative research, the sample size may appear small, but since generalisability was not the focus of our work, the themes identified in our work are still a valuable starting point that merits further investigation. However, the scoping review provides an overview of the topic under consideration, including the gaps in the literature.

## Conclusion

Across all shift patterns, nurses describe how they strive to deliver high quality of care and resort to various mechanisms to cope with an ever-changing and demanding work environment. From the current literature, it is evident that shift patterns are often organised in ways that are detrimental to nurses’ health and wellbeing, to their job performance, and consequently, to the patient care they provide. Our findings highlight a number of factors that may be important in influencing nurses’ choice of shift patterns and the resulting outcomes for quality of care and the staff themselves.

While important issues such as individual differences, accommodating preferences and the need to manage fatigue are highlighted by these findings, it is not clear how best to organise shifts. The mixed findings on experience and preference for both long shifts and night work are in contrast with observational studies that show long shifts to be associated with adverse outcomes. Further research should explore the extent to which nurses’ preferences are considered when choosing or being imposed shift work patterns. Research should also strive to better describe and address the constraints nurses face when it comes to choice around shift patterns, including childcare or any other caring responsibilities, as well as individual factors such as age, with the aim to consider these constraints when restructuring shift patterns.

## Supporting information

S1 Checklist PRISMA-P 2015 checklist. (DOCX)

S1 File (DOCX)
