# Correction: Nurses’ experiences and preferences around shift patterns: A scoping review

**DOI:** 10.1371/journal.pone.0270446

**Published:** 2022-06-21

**Authors:** Ourega-Zoé Ejebu, Chiara Dall’Ora, Peter Griffiths

In the Funding statement, the funding recipient is listed incorrectly. The correct funding statement is: CDO received funding from NIHR Applied Research Collaboration Wessex; https://www.arc-wx.nihr.ac.uk/research-areas/workforce-and-health-systems/nursing-shift-patterns-in-acute-community-and-mental-health-hospital-wards/. The funders had no role in study design, data collection and analysis, decision to publish, or preparation of the manuscript.
