# Supplementary material for: Nurses’ experiences and preferences around shift patterns: A scoping review
Source: PLoS One. 2021 Aug 16;16(8):e0256300. doi: 10.1371/journal.pone.0256300 (PMC8367008; doi:10.1371/journal.pone.0256300)
Supplement: S1 File — (DOCX) [file pone.0256300.s002.docx]

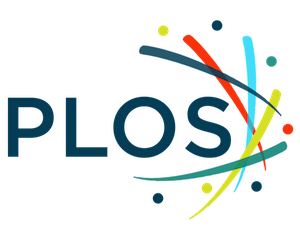


Study Protocol for a Scoping Review

Nurses’ experiences and preferences around shift patterns: A scoping review

Funding

NIHR Applied Research Collaboration Wessex, <https://clahrc-wessex.nihr.ac.uk/>

The funders had no role in the study design, data collection and analysis, decision to publish, or preparation of the manuscript.

Competing interests

The authors have declared that no competing interests exist.

Authors

- Ourega-Zoé Ejebu, National Institute for Health Research, Applied Research Collaboration Wessex School of Health Sciences, University of Southampton, Southampton, UK, [o.ejebu@soton.ac.uk](mailto:o.ejebu@soton.ac.uk)
- Chiara Dall’Ora**^*^** **(Corresponding author)**, National Institute for Health Research, Applied Research Collaboration Wessex School of Health Sciences, University of Southampton, Southampton, UK, [C.Dallora@soton.ac.uk](mailto:C.Dallora@soton.ac.uk)
- Peter Griffiths, National Institute for Health Research, Applied Research Collaboration Wessex School of Health Sciences, University of Southampton, Southampton, UK, [peter.griffiths@soton.ac.uk](mailto:peter.griffiths@soton.ac.uk)

Data availability

All data are fully available without restriction

Abstract

**Objective**: To explore the evidence on nurses’ experiences and preferences around shift patterns in the international literature.

**Data sources**: Electronic databases (CINHAL, MEDLINE and Scopus) were searched to identify primary studies up to April 2021.

**Methods**: Papers reporting qualitative or quantitative studies exploring the subjective experience and/or preferences of nurses around shift patterns were considered, with no restrictions on methods, date or setting. Key study features were extracted including setting, design and results. Findings were organised thematically by key features of shift work.

**Results**: 30 relevant papers were published between 1993 and 2021. They contained mostly qualitative studies where nurses reflected on their experience and preferences around shift patterns. The studies reported on three major aspects of shift work: shift work *per se* (i.e. the mere fact of working shift), shift length, and time of shift.

Across all three aspects of shift work, nurses strive to deliver high quality of care despite facing intense working conditions, experiencing physical and mental fatigue or exhaustion. Preference for or adaptation to a specific shift pattern is facilitated when nurses are consulted before its implementation or have a certain autonomy to self-roster. Days off work tend to mitigate the adverse effects of working (short, long, early or night) shifts. How shift work and patterns impact on experiences and preferences seems to also vary according to nurses’ personal characteristics and circumstances (e.g. age, caring responsibilities, years of experience).

**Conclusions**: Shift patterns are often organised in ways that are detrimental to nurses’ health and wellbeing, their job performance, and the patient care they provide. Further research should explore the extent to which nurses’ preferences are considered when choosing or being imposed shift work patterns. Research should also strive to better describe and address the constraints nurses face when it comes to choice around shift patterns.

Introduction

**Rationale**

Shift work is an established feature of working life for many hospital nurses, who work to provide 24-hour healthcare. Several directives and regulations influence how shift work is organised, including the European Working Time Directive of 2003^1^ and the US Fair Labor Standards Act of 1938^2^. Such directives limits the maximum number of weekly hours or regulate the frequency of work breaks. Notwithstanding such regulations, shift work can be organised in a variety of ways, in terms of shift length, overtime, weekly hours, rotating and/or permanent schedules. How shift patterns are organised play a key role in factors influencing nurses’ wellbeing and performance, as well as patient outcomes and health systems’ productivity^3^.

For instance, shift work may require nurses to work overnight causing adverse health effects, such as increased sleepiness at the end of the shift ^4^ or disturbed sleep^5^. Shift work schedules can also have unintended consequences depending on whether they are rotating or permanent. Working permanent night shifts is associated with higher long-term sickness absence rates in comparison to day-shifts only ^6^. In the same vein, working as part of a rotating schedule is associated with increased levels of acute fatigue ^7^, errors ^8^ and higher risks of alcohol consumption ^9^. These factors can in turn jeopardise the quality of care.

While a three-shift pattern with two 8-hour day shifts and a night shift remains common, long shifts of 12 hours or more as part of a two-shift system have become standard in many countries including Ireland, Poland, the USA and increasingly in the UK ^10,11^. Despite a number of claims that a two-shift system is more efficient, there is no evidence of productivity gain when working long shifts ^12^ and job dissatisfaction is higher among nurses working long shifts ^11,13,14^. Working long shifts is also associated with nurses reporting reduced educational opportunities, fewer opportunities to discuss patient care ^15^, increased delayed or missed care ^16^ and higher (pneumonia) mortality rates ^17^ in comparison to shorter shifts. Nurses working long shifts are also more likely to experience burnout and report intention to leave in comparison to their counterparts ^13,14^.

Despite such adverse outcomes, some literature suggest certain nurses prefer working long shifts, as evidenced by their higher job and schedule satisfaction, as well as their lower emotional exhaustion level ^18^. Preference for long shifts is also attributed to improved work-life balance ^19^, higher number of days off and opportunities for greater continuity of care ^20^. However, the mechanisms explaining such preferences, how nurses experience these shift patterns, and how these shift patterns interact with other aspects of their life remains unclear.

The evidence on nurses’ subjective experience and preferences around shift patterns has not been summarised, as quantitative studies reporting associations dominate the field. In these studies, adverse experiences are indirectly inferred from (for example) reported associations between shift patterns and burnout. The purpose of such quantitative studies is generally not to capture nurses’ perspectives.

Yet, insights from nurses’ perspective are key to better understand mechanisms of preference and choice around shift patterns. Studies where nurses’ perspectives are directly obtained (rather than inferred by the researcher) could shed further light on the contradictions arising from the quantitative body of evidence. Nursing staff form the largest group in the health workforce, and comprehending their experience and preferences around shift patterns is key to effectively improve nursing working conditions, enhance nurses’ job satisfaction and increase quality of care.

**Objectives**

The aim of this review is to examine and summarise the extent, range and nature of research activity on nurses’ subjective experience and preferences around shift patterns. We conducted a scoping review ^21,22^, aiming to summarise existing evidence and highlight gaps. The PI(C)O process is as follows: Participants (nursing staff), comparators (patterns of shifts), and outcomes (experience, preference, choice)

Materials and Methods

**Eligibility criteria**

We limited our search to studies with an English language abstract but there was no restriction on the publication date. We only included papers that contained explicit comments or views as reported by nursing staff, whilst excluding papers that made indirect inferences. Papers that were not specific to nursing, as well as news articles and opinions were excluded from the scoping review.

**Information sources**

We searched CINAHL, Medline and Scopus with terms pertaining to nurses’ experience and preferences around shift patterns up to April 2021.

**Search strategy**

| **CINHAL (EBSCO) (N=1,225)**  S1 shift work  S2 work schedule  S3 shift pattern  S4 shift length  S5 S1 OR S2 OR S3 OR S4  S6 “nurse”  S7 health professionals  S8 S6 OR S7  S9 S5 AND S8  S10 “impact”  S11 “effect”  S12 “affect”  S13 “perception”  S14 “experience”  S15 “reaction”  S16 “prefer*”  S17 S10 OR S11 OR S12 OR S13 OR S14 OR S15 OR S16  S18 S9 AND S17 Limiters: English  S19 S9 AND S16 Limiters: English – Research Article |
| --- |
| **Medline (Ovid) (N= 1,054)**  1. (shift adj4 work*).mp. [mp=title, abstract, original title, name of substance word, subject heading word, floating sub-heading word, keyword heading word, organism supplementary concept word, protocol supplementary concept word, rare disease supplementary concept word, unique identifier, synonyms]  2. work* schedule.mp.  3. shift pattern*.mp.  4. shift length.mp.  5. "Personnel Staffing and Scheduling"/  6. (shift or schedule).mp. [mp=title, abstract, original title, name of substance word, subject heading word, floating sub-heading word, keyword heading word, organism supplementary concept word, protocol supplementary concept word, rare disease supplementary concept word, unique identifier, synonyms]  7. 5 and 6  8. 1 or 2 or 3 or 4 or 7  9. nurse*.mp.  10. health professional.mp.  11. 9 or 10  12. impact.mp.  13. effect.mp.  14. affect.mp. or Affect/  15. perception.mp. or Perception/  16. experience.mp.  17. reaction*.mp.  18. preference.mp.  19. 12 or 13 or 14 or 15 or 16 or 17 or 18  20. 8 and 11 and 19  21. 20 and “Journal Article” [Publication Type] |
| **Scopus (Elsevier) (N=1,127)**  ( ( TITLE-ABS-KEY ( "shift work" ) ) OR ( TITLE-ABS-KEY ( "work schedule" ) ) OR ( TITLE-ABS-KEY ( "shift pattern" ) ) OR ( TITLE-ABS-KEY ( "shift length" ) ) ) AND ( ( TITLE-ABS-KEY ( "nurse" ) ) OR ( TITLE-ABS-KEY ( "health professional" ) ) ) AND ( ( TITLE-ABS-KEY ( "impact" ) ) OR ( TITLE-ABS-KEY ( "effect" ) ) OR ( TITLE-ABS-KEY ( "affect" ) ) OR ( TITLE-ABS-KEY ( "perception" ) ) OR ( TITLE-ABS-KEY ( "experience" ) ) OR ( TITLE-ABS-KEY ( "reaction" ) ) OR (TITLE-ABS-KEY(“prefer*”) ) ) AND ( LIMIT-TO ( LANGUAGE , "English" ) ) AND ( LIMIT-TO ( DOCTYPE , "ar" ) ) |

**Study Records**

All papers included in the review were stored in Endnote ®. OE applied the inclusion/exclusion criteria to screen all titles and abstracts, after which CDO and PG reviewed the selections. We extracted publication date, respondents’ roles (i.e. registered vs unregistered nurse), working place and shift characteristics for each study. Using Excel®, we tabulated a spreadsheet where results were recorded and organised by themes based on the authors’ findings. Using an iteration process by re-reading and analysing our results, we separated results based on the advantages and disadvantages of various shift patterns. Results reported on three core aspects of shift work, shift work *per se*, shift length and time of shift. Ethical approval was not required for the scoping review.

Discussion

Following the scoping review process, we did not conduct a systematic appraisal of the qualities of studies ^21^. Whilst this approach widens the scope of studies included in the review, it may also bias the conclusion of our findings as the strength of the evidence is not being assessed. However, the scoping review provides an overview of the topic under consideration, including the gaps in the literature.

Authors’ contributions

CDO and PG had the original idea for this project and obtained funding for researcher salaries. All authors contributed to the development of the scoping review. All authors agreed that the sample of articles selected for full-text review were relevant for the research question. OE extracted data from relevant studies and met regularly with CDO and PE to discuss findings. All authors reviewed each successive draft and contributed to the final paper. CDO and PG are responsible for the overall content as guarantors. The corresponding author attests that all listed authors meet authorship criteria and that no others meeting the criteria have been omitted.

Acknowledgements

Not applicable

Supporting Information

PRISMA-P checklist

References

1. European Commission. *Working Conditions - Working Time Directive*. <https://ec.europa.eu/social/main.jsp?catId=706&langId=en&intPageId=205> (Accessed 16 July 2021).

2. U.S. Department of Labor Wage and Hour Division. *Fair Labor Standards Act of 1938*; 2011.

3. Dall'Ora C, Ball J, Recio-Saucedo A, et al. Characteristics of shift work and their impact on employee performance and wellbeing: A literature review. *International Journal of Nursing Studies* 2016;57:12-27.

4. Geiger-Brown J, Rogers VE, Trinkoff AM, et al. Sleep, sleepiness, fatigue, and performance of 12-hour-shift nurses. *Chronobiology International* 2012;29(2):211-19.

5. Kecklund G, Axelsson J. Health consequences of shift work and insufficient sleep. *BMJ* 2016;355:i5210.

6. Dall’Ora C, Ball J, Redfern OC, et al. Night work for hospital nurses and sickness absence: a retrospective study using electronic rostering systems. *Chronobiology International* 2020:1-8.

7. Han K, Trinkoff AM, Geiger-Brown J. Factors associated with work-related fatigue and recovery in hospital nurses working 12-hour shifts. *Workplace Health & Safety* 2014;62(10):409-14.

8. Niu SF, Chu H, Chen CH, et al. A comparison of the effects of fixed- and rotating-shift schedules on nursing staff attention levels: a randomized trial. *Biological Research for Nursing* 2013;15(4):443-50.

9. Trinkoff AM, Storr CL. Work schedule characteristics and substance use in nurses. *American Journal of Industrial Medicine* 1998;34(3):266-71.

10. Griffiths P, Dall'Ora C, Simon M, et al. Nurses' shift length and overtime working in 12 European countries: the association with perceived quality of care and patient safety. *Medical Care* 2014;52(11):975-81.

11. Ball J, Day T, Murrells T, et al. Cross-sectional examination of the association between shift length and hospital nurses job satisfaction and nurse reported quality measures. *BMC Nursing* 2017;16(1):26.

12. Griffiths P, Dall'Ora C, Sinden N, et al. Association between 12-hr shifts and nursing resource use in an acute hospital: Longitudinal study. *Journal of Nursing Management* 2019;27(3):502-08.

13. Dall'Ora C, Griffiths P, Ball J, et al. Association of 12 h shifts and nurses' job satisfaction, burnout and intention to leave: findings from a cross-sectional study of 12 European countries. *BMJ Open* 2015;5(9):e008331.

14. Stimpfel AW, Sloane DM, Aiken LH. The longer the shifts for hospital nurses, the higher the levels of burnout and patient dissatisfaction. *Health Affairs* 2012;31(11):2501-09.

15. Dall'Ora C, Griffiths P, Emmanuel T, et al. 12-hr shifts in nursing: Do they remove unproductive time and information loss or do they reduce education and discussion opportunities for nurses? A cross-sectional study in 12 European countries. *Journal of Clinical Nursing* 2020;29(1-2):53-59.

16. Dall'Ora C, Griffiths P, Redfern O, et al. Nurses' 12-hour shifts and missed or delayed vital signs observations on hospital wards: retrospective observational study. *BMJ Open* 2019;9(1):e024778.

17. Trinkoff AM, Johantgen M, Storr CL, et al. Nurses' work schedule characteristics, nurse staffing, and patient mortality. *Nursing Research* 2011;60(1):1-8.

18. Stone PW, Du Y, Cowell R, et al. Comparison of nurse, system and quality patient care outcomes in 8-hour and 12-hour shifts. *Medical Care* 2006;44(12):1099-106.

19. Day G. Is there a relationship between 12-hour shifts and job satisfaction in nurses. *Alabama Nurse* 2004;31(2):11-12.

20. Ball J, Dall'Ora C, Griffiths P. The 12-hour shift: Friend or foe? *Nursing Times* 2015;111(6):12-14.

21. Arksey H, O'Malley L. Scoping studies: towards a methodological framework. *International Journal of Social Research Methodology* 2005;8(1):19-32.

22. Peters MDJ, Marnie C, Tricco AC, et al. Updated methodological guidance for the conduct of scoping reviews. *JBI Evidence Synthesis* 2020;18(10).
